# Contextual influence of mammalian macro-autophagy in virus-bacteria coinfected cell phenotypes

**DOI:** 10.1371/journal.ppat.1011625

**Published:** 2023-09-21

**Authors:** Christophe Viret, Aude Lavedrine, Guénaëlle Lamiral, Aurore Rozières, Mathias Faure

**Affiliations:** CIRI, Centre International de Recherche en Infectiologie, Université de Lyon, Inserm U1111, Université Claude Bernard Lyon 1, CNRS, UMR5308, ENS de Lyon, Lyon, France; Tufts Univ School of Medicine, UNITED STATES

## Introduction

It is widely recognized that viral infections can substantially impact host resistance to secondary bacterial infections. For example, pulmonary influenza infection can be associated with subsequent infection with bacteria such as *Streptococcus pneumoniae*, *Streptococcus pyogenes*, or *Haemophilus influenzae* leading to increased susceptibility to severe pneumonia [1,2]. More recently, some patients with Coronavirus Disease 2019 (COVID-19) were found susceptible to natural coinfection with *Streptococcus aureus*, *S*. *pneumoniae*, or *H*. *influenzae*, or to various nosocomial bacterial infections in the context of prolonged hospitalization [3]. While great advances are being made in understanding the conditions and consequences related to virus-bacteria coinfection at the level of tissues and organisms (cell damages, immune cell infiltration, proinflammatory cytokine production, dysbiosis) [2,4], our knowledge of the mechanistic changes that can take place at the cellular level remains very limited. Autophagy is a degradative pathway important for cell homeostasis but also an important cell autonomous defense mechanism against intracellular pathogens. Here, we focus on the few available studies that address the question of the influence of virus infection-associated changes in the autophagic status of host cells on the outcome of secondary bacterial infections at the cell level. This is an emerging field of study that, in the future, may contribute to our understanding of the biology of virus/bacteria coinfections at large.

### Autophagy in cell-autonomous antimicrobial defense

**Fig 1:**
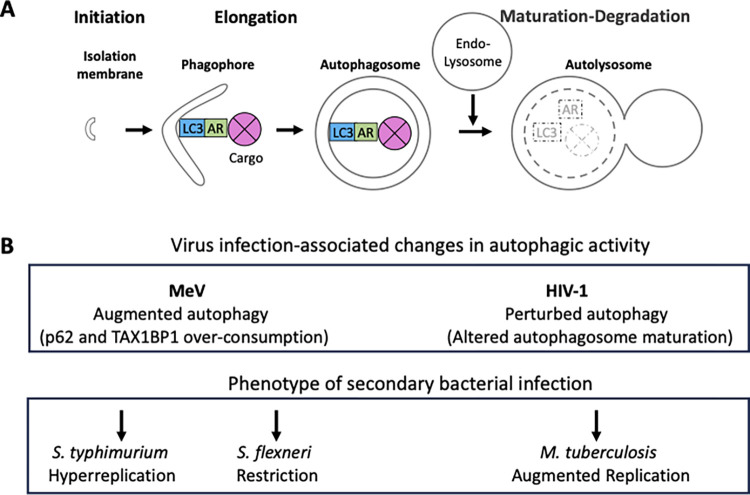
Proviral changes in the autophagy status of infected cells differentially affect the outcome of secondary bacterial infection at the cellular level. (A) A simplified representation of the ordered steps that constitute the autophagy flux (see main text) (AR: autophagy receptors). (B) Summary of available observations on the outcome of secondary bacterial infection of measles virus-infected epithelial cells (*Salmonella Typhimurium or Shigella flexneri*) or HIV-1-infected macrophages (*Mycobacterium tuberculosis*).

Macro-autophagy (hereafter autophagy) is a potent pathway for the degradation and recycling of mis-formed and deleterious cytosolic components through their targeting toward the lysosomal degradation system. Substrates for autophagic degradation are sequestered within de novo-formed autophagic membranes (initiation-elongation) that evolve into double-membrane vesicles called autophagosomes that latter fuse with acidic endo-lysosomal vesicles (maturation), an ordered process named the autophagy flux [5] (Fig 1A). Autophagic degradation is selective when so-called autophagy receptors specifically connect defined substrates (ubiquitinated or not) to the autophagy machinery via anchoring factors such as LC3 [6]. For example, mitophagy is the form of autophagy that selectively degrades dysfunctional mitochondria by engaging particular receptors. Besides participating in cell homeostasis maintenance, selective autophagy constitutes an important cell-autonomous defense mechanism against microbes able to invade the cytosol, a phenomenon globally referred to as xenophagy. Autophagy can thus restrain the cytosolic growth of various types of invading bacteria as well as the replication of multiple viruses that are prototypic intracellular parasites (virophagy). Indeed, autophagy can be so efficient in doing so that most microbes have evolved means to evade, oppose, or even manipulate autophagy to their benefit [7,8].

### Viral infection-associated remodeling of the autophagy machinery can determine the outcome of secondary bacterial infection in epithelial cells

Some viral infections can trigger a vigorous and complete autophagy flux in epithelial cells that indeed promotes optimal viral replication. Such observations naturally raised the question of whether such virus-infected cells could become particularly resistant to secondary infection by bacteria that are normally sensitive to targeting and degradation by autophagy. Recent data indicate that the answer to this question can vary depending on the bacteria involved, and in some instance, be counterintuitive. Measles virus (MeV) infection induces a marked and sustained autophagic activity that promotes its replication in epithelial cells [9]. In contrast, *Salmonella enterica Serovar Typhimurium* (*S*. *typhimurium*) replication can be restrained by the autophagy machinery [7]. Remarkably, the marked autophagic activity induced by MeV has no restrictive effect on *S*. *typhimurium* secondary infection [10]. Instead, the bacterium hyper-replicates in MeV-infected cultures as a result of its selective multiplication within multinucleated epithelial cells. *S*. *typhimurium* can reach the cytosol and be subjected to ubiquitination but fails to be efficiently targeted to developing autophagic membranes due to insufficient amounts of SQSTM1/p62 and TAX1BP1/T6BP autophagy receptors that are typically engaged into anti-Salmonella selective autophagy and are in fact actively consumed by MeV-exacerbated autophagy. This is in contrast with *Shigella flexneri* that normally takes advantage of SQSTM1/p62 to promote its metabolism and optimal replication and therefore, become restricted in MeV-infected syncytia. These observations, which hold in primary human epithelia cells, indicate that the virus-imposed remodeling of the autophagy machinery can be instrumental in determining the fate of bacterial coinfection and that such a fate is greatly determined by the nature of the functional relationship that evolved between a given bacterium and autophagy factors [10]. Whether MeV infection-associated remodeling of the autophagy machinery can contribute to secondary bacterial infection (*S*. *pneumoniae*, *S*. *aureus*, *H*. *influenzae*) that can occur in patients with measles and are known to promote severe pneumonia or laryngotracheobronchitis [11] remains unknown.

### Viral infection-associated alteration of the autophagy flux can promote bacterial survival/growth in macrophages

Opportunistic bacterial infection is a frequent complication in human immunodeficiency virus type 1 (HIV-1)-infected patients and can participate in the pathology-associated mortality. A large fraction of HIV-1-infected patients harbor coinfection with *Mycobacterium tuberculosis*. Until recently, a possible role for HIV-1-associated autophagy modulation in opportunistic bacterial infection has not been studied at the cellular level. The conditions of coinfection have been examined by using macrophages as these cells can be efficiently infected by both pathogens. HIV-1 infection can indeed alter the capacity of macrophages to control the growth of several invading mycobacteria, and in particular of *M*. *tuberculosis*, leading to compromised macrophage survival [12]. Besides a negative regulation of phagocytosis, HIV-1 pro-mycobacterial effect also results from an altered autophagosome maturation and in both cases, the viral factor Nef was found involved [13,14]. More specifically, Nef interacts and interferes with the autophagy factor beclin-1 that, along with UVRAG, VPS34, and VPS15 autophagy factors, participate in the so-called Class III PI3K complex II to positively regulate autophagosome maturation [15]. This interference renders HIV-1 more resistant to autophagic degradation. Thus, changes in the autophagic status of HIV-1-infected macrophages both benefit to the virus and render host cells more permissive to *M*. *tuberculosis* replication.

### Pro-bacterial consequences of virus-imposed autophagy modifications are druggable

A common characteristic of HIV-1 patients is a lowered level of active vitamin D, a feature associated with higher risk for *M*. *tuberculosis* infection. A supply in vitamin D3 at physiological levels was found to reduce HIV-1 replication through autophagy induction in macrophages [16]. In HIV-1/*M*. *tuberculosis-*coinfected macrophages, vitamin D3 inhibits the replication of both pathogens through the induction of cathelicidin antimicrobial peptide (CAMP) that promotes autophagosome and phagolysosome biogenesis [17]. Thus, as the autophagy flux is sensitive to pharmacological interventions, the possibility to attenuate bacteria replication in virus/bacteria coinfected cells by drugs delivery may be of therapeutic interest. Another way to stimulate anti-mycobacterial autophagy in HIV-1/mycobacteria-coinfected cells is to reverse the HIV-1-induced inhibition of the autophagy flux with Trehalose, a naturally occurring disaccharide that activates autophagy. Trehalose-rescued autophagic degradation of *M*. *tuberculosis* depends on the nuclear translocation of the transcription factor TFEB that positively regulate genes of both the autophagy pathway and lysosome homeostasis. In molecular terms, Trehalose recruits the class I PI3Kinase PIKFYVE to generate phosphatidylinositol 3,5-biphosphate leading to lysosomal Ca^2+^ release and TFEB nuclear translocation [18]. Hence, pathogen replication in HIV-1/*M*. *tuberculosis-*coinfected macrophages appears sensitive to drug-mediated activation of the autophagy machinery. However, pharmacological agents able to stimulate autophagy must be considered with caution as it was observed that Rapamycin/Torin1-mediated mTORC1 inhibition indeed enhances *M*. *tuberculosis* replication in HIV-1-coinfected macrophages [19].

### Concluding remarks

The available data indicates that the outcome of virus/bacteria coinfection can be differentially impacted by changes in the autophagic status of mammalian host cells depending on the replication cycle of the pathogens involved and the intricate relationship that evolved between these pathogens and the autophagy machinery (Fig 1B). Of note, autophagy alterations associated with viral infection can translate in bacterial hyper replication in both epithelial and professional immune cells that may then represent bacterial reservoirs. Pharmacological intervention aiming at controlling pathogen replication through modulation of the autophagic activity appears subject to complexity at this stage. As our knowledge of virus/bacteria coinfected cell phenotypes in relation with autophagy is in its infancy, more investigation is clearly needed to explore the influence of the coinfection contexts involved (e.g., virus and bacterial types and strains, cell types, polymorphisms affecting autophagy-related gene), to determine whether robust trends can be identified, and to delineate the contribution of such cellular phenomena to the coinfection phenotype seen at the organism level. For example, elucidating the influence of dysfunctional autophagy in Epstein–Barr virus-*Helicobacter pylori* coinfection is of interest due to its possible role in gastric cancer [20]. Additional studies might also help to determine whether patterns of phenotypes can be identified in relation with the class of microbes involved in coinfection. Another issue relates to the question of whether direct interactions between viruses and bacteria [21] can influence autophagy during coinfection. Investigations on the role of autophagy are also of interest in the context of virus–virus [22], virus–fungi [23], and virus–parasites [24] coinfections. Finally, from a fundamental point of view, it would be interesting to examine how complex is the relationship between autophagy and pathogen coinfection in cells from distant organisms like invertebrates and plants.
